# Dietary Habits of Older Adults in Serbia: Findings From the National Health Survey

**DOI:** 10.3389/fpubh.2021.610873

**Published:** 2021-08-23

**Authors:** Dragana Stosovic, Nadja Vasiljevic, Verica Jovanovic, Andja Cirkovic, Katarina Paunovic, Dragana Davidovic

**Affiliations:** ^1^Centre for Hygiene and Human Ecology, Institute of Public Health of Serbia “Dr Milan Jovanovic Batut”, Belgrade, Serbia; ^2^Institute of Hygiene and Medical Ecology, Faculty of Medicine, University of Belgrade, Belgrade, Serbia; ^3^Centre for Disease Control, Institute of Public Health of Serbia “Dr Milan Jovanovic Batut”, Belgrade, Serbia; ^4^Institute of Medical Statistics and Informatics, Faculty of Medicine, University of Belgrade, Belgrade, Serbia

**Keywords:** age groups, older adults, dietary habits, socioeconomic factors, Serbia

## Abstract

**Objectives:** Adults aged 65 years and older comprise one fifth of the Serbian population. Many of them have multiple, often diet-related comorbidities. We aimed to investigate their dietary habits by comparing them with younger adults' and to determine the relation of the differing ones to demographic, socioeconomic and health factors.

**Methods:** We performed a secondary analysis of 2013 Serbian National Health Survey data on 14,082 adults. Binary logistic regression was used to determine dietary habits associated with older age (≥65 years) compared to younger age (18–64 years) and to assess their independent predictors in older adults.

**Results:** Older adults more often reported everyday breakfast (OR = 2.085, 95%CI = 1.622–2.680) and brown/wholegrain bread consumption (OR = 1.681, 95% CI = 1.537–1.839), while using margarine (OR = 0.578, 95%CI = 0.397–0.839), discretionary salt (sometimes: OR = 0.648, 95%CI = 0.596–0.705, almost always: OR = 0.522, 95%CI = 0.445–0.614) and consuming fish (two or more times a week: OR = 0.465, 95%CI = 0.383–0.566) less frequently than younger adults. This was mainly positively related to urban environment, affluence, higher education and poor health.

**Conclusion:** Using nationally representative data, we found that older adults reported healthier dietary habits compared to younger adults, which requires timely public health action.

## Introduction

Dietary habits are defined as “the habitual decisions of individuals or a group of people regarding what foods they eat” (1). Food choices are influenced by a variety of factors, among which demographic and socioeconomic factors play a significant role and their impact has been extensively investigated (2, 3). When it comes to age, one must consider not only physiological changes that come with older age but also food preferences from lifetime experiences (4). Physiological, psychosocial, and economic factors that appear with aging can be an obstacle to a healthy diet (5). Not only do dietary habits change over a lifetime but so do nutrition requirements, which mainly refer to a lower energy intake, higher protein content, and a higher intake of vitamins and minerals (6). Therefore, many countries developed food-based dietary guidelines aimed specifically at the elderly (7). This is extremely important considering the global growth of the older population and the estimations that every sixth person will be older than 65 years by 2050 (8).

The trend of an aging population is present in Serbia as well, and according to the estimation of the Statistical Office of the Republic of Serbia (9), adults aged 65 years and more comprised 17.8% of the total population in 2013. Also, 47.3% of Serbian elderly men and 65% of elderly women had multiple chronic conditions and diseases (10), often related to diet.

Dietary habits of the older population were rarely targeted compared with the younger population (11–13), and factors related to these differences were not systematically studied. Although most of the evidence provided from cohort studies speaks in favor of healthier dietary habits with increasing age (14, 15), some important studies found different or at least inconclusive results in older age groups (16, 17). Because of the importance and actuality of this issue, further investigation is needed. Therefore, the objective of this study was to investigate dietary habits of older adults (65 years and older) by comparing them with younger adults (18–64 years) from the National Health Survey in Serbia and to assess the relation of the differing ones to demographic, socioeconomic and health factors. We hypothesized that there are specificities in the diet of older adults compared to younger adults and that they are related to demographic, socioeconomic, and health-related factors.

## Methods

### The Serbian National Health Survey

This study is a secondary analysis of the 2013 Serbian National Health Survey data. The survey was conducted in line with the methodology of the European Health Interview Survey wave two (EHIS wave 2) and its Methodological manual (18).

The target population of this survey comprised of individuals 15 years and older, who lived in private households, and whose place of residence was on the territory of Serbia when the survey was carried out. Persons living in collective households and institutions, and the inhabitants of Kosovo and Metohija, were excluded from the survey. This survey also targeted persons aged 7–14 years as a special group, who were not included in this study.

The 2011 Census of Population, Households, and Apartments in the Republic of Serbia was used as a sampling frame, from which a stratified two-stage cluster was chosen. Variables of region and settlement type were used for stratification. The first-stage units were census circles chosen by probability proportional sampling, whereas the households were second-stage units, which were selected with equal selection probability and without repetitions. The sample comprised 6,500 households and 14,623 household members.

The household questionnaire, face-to-face questionnaire, and the self-administered questionnaire were used as the survey instruments. The survey instruments were pretested on a smaller sample so as to adapt and optimize them for the Serbian population. The data collection took place between October 7, 2013, and December 30, 2013. Each of the 68 teams consisted of three members, pre-trained for the survey carryout, of whom one was a health care worker.

The field researchers were obliged to provide the survey participants with hard copies of the Information on the Survey signed by the Minister of Health, the approval of the Ethical Board on conducting the survey, along with the rights of the survey participants and the procedure for filing appeals/complaints in case they believed that their rights were violated. In addition, signed informed consent of enrolment in the survey was obtained from the survey participants.

### Data Analysis and Coding

The data analyzed from this survey included the answers obtained *via* the Household and the Face-to-face questionnaires from the participants aged 18 years and more (*N* = 14,082). We used information on their demographic characteristics, namely gender, age, settlement type and marital status, and socioeconomic status, assessed through educational level and wealth index. The wealth index was calculated based on the household assets (the number of bedrooms per household member, the main material from which the floor, roof, and walls of the house were built, the main source of drinking water, means of sanitation, the energy source used for heating, owning of color television, mobile phone, refrigerator, computer, washing machine, dishwasher, air conditioning, central heating, car, and Internet access), as is described in more detail elsewhere (19).

In addition, we included data on self-reported morbidities in the previous 12 months (asthma, chronic obstructive pulmonary disease, myocardial infarction, angina pectoris, hypertension, stroke, arthrosis, lower back deformity, neck disorder, diabetes, allergy, liver cirrhosis, kidney problems, urinary incontinence, depression, cancer, and hyperlipidemia).

Dietary data were obtained *via* a set of questions within the face-to-face questionnaire. Besides the questions defined by the Methodological manual (18), additional questions on dietary habits were included to follow-up national indicators. The additional questions on dietary habits were defined based on the expert consensus and known diet characteristics in the Serbian population. Hence, the dietary habits that were analyzed included the consumption of breakfast, milk and dairy products, type of preferred bread, type of fat used in food preparation, adding salt to food, fish intake, and fruit and vegetable intake.

The data were coded as follows: study groups- 0 younger adult (18–64 years old) and 1 older adult (65 years and older), gender- 1 man and 2 women, settlement- 1 urban and 2 others [rural areas or a borderline case (18)], wealth index- 1 poorest to 5 richest quintile, marital status- 0 without a partner (not married/not in a relationship, widow/widower, divorced/separated) and 1 married/living with a partner, education status- 1 elementary ( ≤ 8 years), 2 secondary (9–12 years), and 3 higher education (>12 years), and health status- 0 healthy and 1 ill (having at least one morbidity).

When it comes to dietary variables, we merged them according to the observed frequencies and health relevance where appropriate to gain greater informativeness (i.e., brown and wholegrain bread were treated as a single variable due to higher fiber content in comparison to white bread). We coded them in the following manner: frequency of having breakfast in a week- 1 never to 3 every day, consumption of milk and dairy products- 1 never and sometimes (not every day) to 3 two or more cups every day, type of consumed bread- 1 white bread and 0 non-consumption of solely white bread, that is, consumption of other bread types, all types combined and non-consumption of bread (the same coding was used for other answers to this question), type of fat used in food preparation- 1 oil and 0 other answers (the same for the other fat type variables), adding salt to food- 1 never to 3 almost always before tasting, fish intake- 1 never to 3 two times a week and more often, fruit intake- 1 one time to three times a week and less often to 3 one or more times a day; vegetable intake- coding the same as for the fruit. While interpreting the data on dietary habits, we used the current dietary guidelines in the European countries (20) to discuss the observed results.

### Statistical Analysis

Continuous variables were described as means (SD) or medians (interquartile range, IQR) depending on the normality, while categorical variables were presented as frequencies (*n*) and percentages (%).

We calculated the difference in demographic, socioeconomic characteristics, health state, and dietary habits between the two age groups using the Chi-squared test of independence.

We used univariable and multivariable binary logistic regression to determine the predictive dietary habits for older age. *P*-value <0.05 was used for entering the variables into a model in a backward stepwise manner, while the cutoff value for the variance inflation factor (VIF) of >5 was used to identify multicollinearity. In case equal *p*-values were obtained for the bread type variables, the variable with the highest odds ratio (OR) was included in the multivariable analysis. The dietary habits from the final model were consecutively recoded as dummy variables and we performed univariable binary logistic regression to assess their demographic and other independent predictors in the population of older adults.

The data were analyzed using the SPSS statistical software, version 19.0. A significance level of 0.05 was used throughout.

## Results

This study included 14,082 participants (7,595 women), of whom one-fourth belonged to older adults. Overall, the median age was 52 years (IQR 29), while by age groups, it was 73 (IQR 9) and 44 (IQR 24) years, respectively. The study participants predominantly lived in urban settlements (56.4%) and most of them had a partner (64%) (Table 1). Approximately half of them had secondary education levels, while the wealth index was mainly equally distributed with a slight predominance of the poorest quintile (22.4%). In 59.2% of the study participants, the presence of at least one morbidity was noted. There were significantly more women, non-urban dwellers, persons without a partner, and with at least one morbidity among older adults. On the other hand, younger adults were significantly more educated and wealthier.

**Table 1:** Demographic, socioeconomic, and health state characteristics of the study participants by age groups.

**Variable**	**All participants *N* = 14,082**	**Age groups (years)**, ***n*****(%)**		***p-*value^a^**
		**18–64 *n* = 10,542 (74.9)**	**≥65 *n* = 3,540 (25.1)**	
**Gender**, ***n*****(%)**
Male	6,487 (46.1)	4,959 (47)	1,528 (43.2)	<0.001
Female	7,595 (53.9)	5,583 (53)	2,012 (56.8)	
**Type of settlement**, ***n*****(%)**
Urban	7,942 (56.4)	6,039 (57.3)	1,903 (56.4)	<0.001
Other	6,140 (43.6)	4,503 (42.7)	1,637 (45.2)	
**Marital status**, ***n*****(%)**
Married/living with a partner	9,013 (64)	7,048 (66.9)	1,965 (55.5)	<0.001
Without a partner	5,069 (36)	3,494 (33.1)	1,575 (44.5)	
**Education status**, ***n*****(%)**
Elementary	4,137 (29.4)	2,181 (20.7)	1,956 (55.3)	<0.001
Secondary	7,649 (54.3)	6,545 (62.1)	1,104 (31.2)	
Higher	2,296 (16.3)	1,816 (17.2)	480 (13.6)	
**Wealth index**, ***n*****(%)**
Poorest	3,156 (22.4)	1,968 (18.7)	1,188 (33.6)	<0.001
Second	3,021 (21.5)	2,234 (21.2)	787 (22.2)	
Middle	2,815 (20)	2,184 (20.7)	631 (17.8)	
Fourth	2,629 (18.7)	2,043 (19.4)	586 (16.6)	
Richest	2,461 (17.5)	2,113 (20)	348 (9.8)	
**Health state**, ***n*****(%)**
Healthy	5,740 (40.8)	5,342 (50.7)	398 (11.2)	<0.001
Ill (with at least one morbidity)	8,342 (59.2)	5,200 (49.3)	3,142 (88.8)	

*National Health Survey, Serbia, 2013*.

a *Chi-square test*.

The dietary habits of the study participants, altogether and according to their age group, are shown in Table 2. Most of the study participants had breakfast every day but did not consume milk and dairy products daily. They preferred white bread and mostly used oil during food preparation. More than half reported never adding salt to food. Furthermore, they predominantly ate fish <2 times a week, whereas fruit and vegetables were consumed mainly one or more times a day.

**Table 2:** Dietary habits of the study participants by age groups.

**Variable**	**All participants *N* = 14,082**	**Age groups (years)**, ***n*****(%)**		***p*-value^a^**
		**18–64, *n* = 10,542 (74.9)**	**≥65, *n* = 3,540 (25.1)**	
**Frequency of having breakfast in a week**, ***n*****(%)**
Never	499 (3.5)	423 (4.0)	76 (2.1)	<0.001
Sometimes	2,473 (17.6)	2,097 (19.9)	376 (10.6)	
Everyday	11,110 (78.9)	8,022 (76.1)	3,088 (87.2)	
**Consumption of milk and dairy products**, ***n*****(%)**
Never and sometimes, not every day	6,907 (49)	5,247 (49.8)	1,660 (46.9)	<0.001
A cup every day	5,643 (40.1)	4,182 (39.7)	1,461 (41.3)	
≥2 cups every day	1,532 (10.9)	1,113 (10.6)	419 (11.8)	
**Type of consumed bread**, ***n*****(%)**
White	8,376 (59.5)	6,424 (60.9)	1,952 (55.1)	<0.001
Brown and wholegrain	3,070 (21.8)	2,037 (19.3)	1,033 (29.2)	
All types	2,510 (17.8)	1,964 (18.6)	546 (15.4)	
Non-consumption of bread	126 (0.9)	117 (1.1)	9 (0.3)	
**Type of fat for food preparation**, ***n*****(%)**
Lard, butter	3,701 (26.4)	2,786 (26.5)	915 (25.9)	0.086
Margarine	195 (1.4)	160 (1.5)	35 (1.0)	
Oil	10,039 (71.5)	7,484 (71.2)	2,555 (72.4)	
Non-use of fat	98 (0.7)	76 (0.7)	22 (0.6)	
**Adding salt to food**, ***n*****(%)**
Never	7,475 (53.1)	5,260 (49.9)	2,215 (62.6)	<0.001
When the food is not salty	5,396 (38.3)	4,274 (40.5)	1,122 (31.7)	
Almost always before tasting	1,211 (8.6)	1,008 (9.6)	203 (5.7)	
**Fish intake**, ***n*****(%)**
Never	738 (5.3)	481 (4.6)	257 (7.3)	<0.001
<2 times a week	11,589 (82.5)	8,697 (82.7)	2,892 (81.9)	
≥2 times a week	1,715 (12.2)	1,333 (12.7)	382 (10.8)	
**Fruit intake**, ***n*****(%)**
1–3 times a week and less often	3,745 (26.6)	2,870 (27.2)	875 (24.7)	<0.001
4–6 times a week	3,777 (26.8)	2,864 (27.2)	913 (25.8)	
≥1 time a day	6,560 (46.6)	4,808 (45.6)	1,752 (49.5)	
**Vegetable intake**, ***n*****(%)**
1–3 times a week and less often	1,849 (13.1)	1,370 (13.0)	479 (13.5)	0.102
4–6 times a week	4,050 (28.8)	2,992 (28.4)	1,058 (29.9)	
≥1 time a day	8,183 (58.1)	6,180 (58.6)	2,003 (56.6)	

*National Health Survey, Serbia, 2013*.

a *Chi-square test*.

In comparison to younger adults, older adults more often had everyday breakfast and one or more cups of milk and dairy products daily. Furthermore, they consumed brown and wholegrain bread more often compared with the younger age group, who were more frequent consumers of white and all types of bread. Besides, younger adults added salt to food more often, either when it was not salty or before tasting it. However, older adults consumed fish significantly less often. Moreover, 49.5% of older adults consumed fruit one or more times a day, whereas 45.6% of younger adults consumed it that often (*p* <0.001). There were no significant differences in terms of the type of fat used in food preparation and vegetable intake between the two age groups.

The study participants who had breakfast every day were 2.14 times more likely to be 65 years and older than the ones who skipped this meal (Table 3). On the other hand, people who consumed one and more cups of milk and dairy products per day had 10.4 and 19% higher odds to be in the older adults age group than the people who consumed them less often, respectively. When it comes to bread type, people who consumed white, all types, or no bread whatsoever were less likely to belong to older adults, whereas the odds of being an older adult were 72% higher in people who consumed brown and wholegrain bread. Adults who used margarine in food preparation were 35% less likely to be in the older age group. Adding salt to the food occasionally or always decreased the odds of being an older adult by 37.7 and 52.2%, respectively, compared with those not using salt at all. The same goes for fish, where these odds gradually decreased with the higher frequency of consumption.

**Table 3:** Dietary habits associated with older age group.

**Variable**	**Univariable analysis^a^**		**Multivariable analysis^a^**	
	**OR (95% CI)**	***p***	**OR (95% CI)**	***p***
**Frequency of having breakfast in a week**
Never	Ref.		Ref.	
Sometimes	0.998 (0.764–1.304)	0.988	0.975 (0.744–1.280)	0.858
Every day	2.143 (1.672–2.745)	<0.001	2.085 (1.622–2.680)	<0.001
**Consumption of milk and dairy products**
Never and sometimes, not every day	Ref.			
A cup every day	1.104 (1.018–1.198)	0.017		
≥2 cups every day	1.190 (1.050–1.349)	0.006		
**Type of consumed bread**
White	0.788 (0.730–0.851)	<0.001		
Brown and wholegrain	1.720 (1.577–1.877)	<0.001	1.681 (1.537–1.839)	<0.001
All types	0.796 (0.718–0.883)	<0.001		
Non-consumption of bread	0.227 (0.115–0.448)	<0.001		
**Type of fat for food preparation**
Lard, butter	0.971 (0.89–1.059)	0.502		
Margarine	0.648 (0.449–0.936)	0.021	0.578 (0.397–0.839)	0.004
Oil	1.061 (0.975–1.156)	0.170		
Non-use of fat	0.861 (0.535–1.387)	0.539		
**Adding salt to food**
Never	Ref.		Ref.	
When the food is not salty	0.623 (0.574–0.677)	<0.001	0.648 (0.596–0.705)	<0.001
Almost always before tasting	0.478 (0.408–0.561)	<0.001	0.522 (0.445–0.614)	<0.001
**Fish intake**
Never	Ref.		Ref.	
<2 times a week	0.622 (0.532–0.728)	<0.001	0.613 (0.521–0.721)	<0.001
≥2 times a week	0.536 (0.444–0.648)	<0.001	0.465 (0.383–0.566)	<0.001
**Fruit intake**
1–3 times a week and less often	Ref.			
4–6 times a week	1.046 (0.940–1.163)	0.410		
≥1 time a day	1.195 (1.089–1.312)	<0.001		
**Vegetable intake**
1–3 times a week and less often	Ref.			
4–6 times a week	1.011 (0.892–1.147)	0.860		
≥1 time a day	0.927 (0.826–1.041)	0.199		

*National Health Survey, Serbia, 2013*.

a *Binary logistic regression*.

The best-fitted model for the older age group included regular breakfast, brown and wholegrain bread, use of margarine in food preparation, adding salt to food, and fish intake (Table 3).

Results of univariable logistic regression of dietary habits as dependent variables and non-dietary variables as independent variables are demonstrated in Table 4. Male gender and middle wealth index were positively related to regular breakfast consumption. Urban dwellers, persons with higher wealth index, and secondary and higher education levels preferred brown and wholegrain bread in their diet. In addition, the wealthiest and the most educated persons used margarine in food preparation. Men, married or persons living with a partner and healthy individuals were more likely to add salt to food consistently. When it comes to fish intake, urban settings, higher wealth quintiles, being married or living with a partner, and increasing education level were positively related to higher weekly intakes.

**Table 4:** Factors associated with dietary habits of older adults in univariate binary logistic regression.

**Variable**	**Everyday breakfast**	**Brown and wholegrain bread**	**Margarine**	**Adding salt almost always**	**Fish intake ≥2 times a week**
			**OR (95% CI)** ***p*** **-value**		
Gender^a^	0.752 (0.613–0.921) 0.006	0.994 (0.859–1.150)0.934	1.283 (0.644–2.556) 0.478	0.423 (0.316–0.568) <0.001	0.844 (0.682–1.045) 0.120
Settlement type^b^	1.000 (0.821–1.219) 0.999	0.537 (0.462–0.623) <0.001	0.604 (0.299–1.217)0.158	1.046 (0.787–1.389)0.758	0.579 (0.464–0.723) <0.001
Wealth index^c^	1.237 (0.948–1.614) 0.117	1.334 (1.091–1.633)0.005	1.071 (0.339–3.385) 0.908	1.124 (0.773–1.633)0.541	1.932 (1.379–2.706) <0.001
	1.394 (1.037–1.873) 0.028	1.692 (1.372–2.088) <0.001	2.157 (0.779–5.976) 0.139	0.838 (0.542–1.295)0.426	2.362 (1.678–3.325) <0.001
	1.303 (0.967–1.755) 0.082	1.318 (1.057–1.645)0.014	1.738 (0.581–5.195) 0.322	0.937 (0.609–1.442)0.767	2.881 (2.058–4.033) <0.001
	1.339 (0.928–1.931) 0.118	1.393 (1.071–1.812)0.013	4.445 (1.643–12.024) 0.003	0.989 (0.592–1.651)0.066	4.224 (2.948–6.053) <0.001
Marital status^d^	1.153 (0.946–1.405) 0.159	1.037 (0.896–1.200)0.624	0.841 (0.432–1.638) 0.611	1.491 (1.110–2.004)0.008	1.351 (1.086–1.680) 0.007
Education status^e^	1.065 (0.855–1.327) 0.575	1.544 (1.313–1.815) <0.001	0.942 (0.398–2.230) 0.942	1.119 (0.820–1.526)0.479	2.020 (1.582–2.580) <0.001
	1.330 (0.965–1.833) 0.081	1.814 (1.468–2.241) <0.001	3.326 (1.546–7.152) 0.002	0.883 (0.561–1.390)0.591	3.242 (2.442–4.304) <0.001
Health state^f^	1.082 (0.797–1.469) 0.612	1.136 (0.899–1.437)0.285	0.983 (0.345–2.799) 0.974	0.525 (0.363–0.760)0.001	0.888 (0.642–1.230) 0.475

*National Health Survey, Serbia, 2013*.

a *Male (referent) vs. female*.

b *Urban (referent) vs. other*.

c *Poorest (referent), second, middle, fourth, richest*.

d *Without a partner (referent) vs. married/ living with a partner*.

e *Low (referent), secondary, higher*.

f *Healthy (referent) vs. ill (having at least one morbidity)*.

## Discussion

This study investigated the dietary habits of Serbian older adults (≥65 years) by comparing them with younger adults (18–64 years) and sought an explanation for the observed differences in socioeconomic and other factors described in the literature.

We found that older adults reported generally healthier dietary habits than younger adults, such as having regular breakfast, opting for brown and wholegrain bread, and avoiding the use of margarine in food preparation, and adding salt to food. The exception was less frequent fish intake. Gender, settlement type, wealth index, marital and education status, and the presence of illness, were all important predictors of the dietary habits that differed between the two age groups.

The findings correspond to the ones derived from the descriptive analysis of the survey data on participants aged 15 years and more (21) but further elaborate and highlight the overall diet of the older Serbian population and the associated factors.

Large national cross-sectional studies have been recognized as a resourceful method to assess dietary intake at different life stages (4); however, most of the existing studies on dietary habits and patterns address age as one of the demographic factors (22), make comparisons among more than two age groups (2, 23), or investigate them solely in the elderly population (24, 25). Moreover, the definition of the elderly often varies interculturally, whereby persons aged 50 years and more are in some cases classified into this category (12, 25). In addition, the methods used for the intake assessment are diverse. Importantly, the findings from existing studies regarding the difference in dietary habits between older adults and the rest of the adult population were mainly accessory and not specifically integrated within their aims. Therefore, we compared the obtained results with somewhat differently designed studies.

Serbian older adults reported having breakfast more regularly than their younger counterparts did, which corresponds to the results derived from the 2005–2016 National Health and Nutrition Examination Survey, whereby adults older than 70 years had reported consuming breakfast in a significantly greater proportion (23). Other studies also confirmed that older adults were less likely to skip this meal (11, 26).

Older adults also opted for bread types with a higher dietary fiber content more often than younger adults, which was also the case among persons belonging to older age groups in the Swedish national dietary survey (27) and nationally representative sample of the Irish population (28). However, the consumption of white bread remains markedly predominant. Given the evidence of health benefits of dietary wholegrain intake in terms of prevention of type 2 diabetes, cardiovascular disease, and colorectal, pancreatic, and gastric cancers, sufficient consumption of whole grains might be a justifiable public health goal (29) in the Serbian population.

Serbian older adults were less likely to use margarine during food preparation, that is, cooking, baking, etc., in this study. On the other hand, margarine contributed to trans-fatty acid intake equally in adults and elderly people in South-eastern Brazil and was the main source of the trans-fatty acids (30). Usually, in Serbia, hard margarine is used for baking, which is proved to be inadequate in terms of fatty acid composition (low in polyunsaturated fatty acids, high in saturated fatty acids, and very high in trans-fatty acids), consequently leading to markedly high atherogenic and thrombogenic indices (31). Although the findings of this study are, thus, promising in terms of health benefits, it is concerning that one-fourth of older adults used lard and butter in food preparation, which is not concordant with the current food-based dietary guidelines in the European countries advising avoidance of animal fat (20).

In this study, older persons were less likely to add salt to food occasionally (OR = 0.65, 95% CI = 0.60–0.70) or nearly always (OR = 0.52, 95% CI = 0.44–0.61). Similarly, in the Argentinian adult population, persons older than 55 years were more likely to avoid frequent/continual use of discretionary salt, that is, in cooking (OR = 0.66) and at the table (OR = 0.67) (32). On the other hand, Brazilians aged 65 years and tended to use more discretionary salt than their younger counterparts (13). A higher salt intake was also noticed in Turkish older adults compared with adults younger than 65 years (33). Moreover, a study estimating the sodium intake in 1990 and 2010 on global, regional and national level reported only small differences within regions and countries when comparing younger adults with older adults (34). Still, findings from a longitudinal study suggest that there is a decrease in absolute salt intake with aging, although it nevertheless exceeds the recommended levels (35). Similarly, in this study, more than one-third of older adults added salt to the food occasionally or always, which is not aligned to the majority of food-based dietary guidelines advising the avoidance of adding salt to food (20). These findings implicate potential targeted health promotion interventions regarding health education in the older Serbian population and the need for further research on sodium intake, including identifying and assessing the contribution of different sources.

Fish consumption was less frequent in older adults compared with younger adults. Similarly, men presented a decrease in fish consumption with increasing age, while there was no difference among women in Brazil (36). On the contrary, Bosnian adults aged 61 years and more consumed significantly more fish than younger study participants (37). Generally speaking, fish consumption varies geographically (3) and fish is not a part of the Serbian traditional diet. Therefore, it is not surprising that the majority of older adults consumed fish <2 times a week.

Observed demographic, socioeconomic, and health disparities could explain these differences to some extent. Namely, favorable dietary habits and avoidance of the harmful ones were mainly related to urban environment, increasing education level, and wealth index, and the presence of an illness, while the influence of gender and marital status was inconsistent. The complexity of gender differences in food choices and dietary habits in terms of dietary components and age categories has already been reported (38). Older persons living in rural areas have additional obstacles toward healthful diet related to that environment, such as lower socioeconomic status and less accessible healthy food (24). Having a partner is predominantly positively associated with healthy eating behavior in the literature (6, 37), while in this study the association is inconclusive. The inverse relation between the prevalence of inadequate nutrient intake and wealth status has already been noted (12). Economic difficulties can affect the dietary habits of older people and, for instance, fish can be viewed upon as a luxurious food item to integrate with diet (39). Higher education coincides with healthier food choices in other studies (22). A possible explanation could be that higher educated individuals also possess better knowledge related to nutrition (40). Finally, the positive relation of a healthier diet with an impaired health state is described in the cross-sectional studies and occurs as a result of limitations to distinguish the cause-effect relationship (2). Therefore, a cross-sectional survey data could suggest an improved diet after the diagnosis in case of findings of healthier intake by individuals diagnosed with chronic diseases compared with those without them (41). An improved diet quality could be related to a stronger protective effect of nutrition knowledge and the use of food labels among patients compared with non-patients.

This study had several limitations. The assessment of dietary habits was semi-quantitative, while it usually includes methods such as 24-h recall or a more detailed food frequency questionnaire. In addition, we did not analyze other factors that might influence dietary habits, one of them being lifestyle habits (physical activity level, smoking, etc.). Moreover, information on how long the participants maintained their dietary habits is missing, as they could have changed their habits due to illness, poor socioeconomic status, or other reasons. However, the methodology was based on the EHIS without major adaptations of the questionnaire (18), so we considered it comparable with the surveys in other countries. Finally, self-reported dietary intakes could be influenced by reporting errors, along with self-reported morbidities.

Nevertheless, this is the first analysis focusing on dietary habits of Serbian older adults based on nationally representative sample data, thus giving an insight into the dietary habits of this population in a Balkan region country. Given the fact that dietary habits are investigated in the population of the same geographical origin, the differences can be observed with minor cultural disparities, in terms of regional, ethnic, and religious variations. This study is also a good cornerstone for further in-depth research of the dietary habits and dietary patterns of Serbian older adults. Further research should comprise a more detailed food frequency questionnaire and/or a 24-h dietary recall and define in precise the duration of adherence to the specific dietary habits, as well as the reasons for a potential change in them.

In conclusion, this study gave an insight on the diet of the population of Serbian older adults and revealed that they had breakfast every day, consumed brown and wholegrain bread, avoided the use of margarine in food preparation, and of discretionary use of salt to a higher extent than younger adults but ate fish less frequently. The observed differences were related to gender, settlement type, wealth index, marital and education status, and the presence of at least one morbidity in a complex way. Overall, Serbian older adults reported healthier dietary habits compared with younger adults, which calls for a timely and targeted public health action.

## Data Availability Statement

The data analyzed in this study is subject to the following licenses/restrictions: Data were available upon the approval of the Ethical board of the Institute of Public Health Dr. Milan Jovanovic Batut. Requests to access these datasets should be directed to kabinet@batut.org.rs.

## Ethics Statement

The studies involving human participants were reviewed and approved by Institute of Public Health of Serbia Dr. Milan Jovanovic Batut. The participants provided their written informed consent to participate in this study.

## Author Contributions

DS and NV made conceptualized and designed the study, they were involved in data analysis, and drafting of the manuscript. VJ and AC were involved in data interpretation and revision of the manuscript. KP and DD edited and revised the manuscript critically. All authors read and approved the final manuscript.

## Conflict of Interest

The authors declare that the research was conducted in the absence of any commercial or financial relationships that could be construed as a potential conflict of interest.

## Publisher's Note

All claims expressed in this article are solely those of the authors and do not necessarily represent those of their affiliated organizations, or those of the publisher, the editors and the reviewers. Any product that may be evaluated in this article, or claim that may be made by its manufacturer, is not guaranteed or endorsed by the publisher.
